# Echocardiographic Image Quality Deteriorates with Age in Children and Young Adults with Duchenne Muscular Dystrophy

**DOI:** 10.3389/fcvm.2017.00082

**Published:** 2017-12-20

**Authors:** Alyssa Power, Sabrina Poonja, Dal Disler, Kimberley Myers, David J. Patton, Jean K. Mah, Nowell M. Fine, Steven C. Greenway

**Affiliations:** ^1^Department of Paediatrics, Alberta Children’s Hospital Research Institute, University of Calgary, Calgary, AB, Canada; ^2^Department of Cardiac Sciences, Libin Cardiovascular Institute of Alberta, University of Calgary, Calgary, AB, Canada

**Keywords:** Duchenne muscular dystrophy, cardiomyopathy, echocardiography, image quality, cardiac magnetic resonance imaging, pediatric cardiology

## Abstract

**Background:**

Advances in medical care for patients with Duchenne muscular dystrophy (DMD) have resulted in improved survival and an increased prevalence of cardiomyopathy. Serial echocardiographic surveillance is recommended to detect early cardiac dysfunction and initiate medical therapy. Clinical anecdote suggests that echocardiographic quality diminishes over time, impeding accurate assessment of left ventricular systolic function. Furthermore, evidence-based guidelines for the use of cardiac imaging in DMD, including cardiac magnetic resonance imaging (CMR), are limited. The objective of our single-center, retrospective study was to quantify the deterioration in echocardiographic image quality with increasing patient age and identify an age at which CMR should be considered.

**Methods:**

We retrospectively reviewed and graded the image quality of serial echocardiograms obtained in young patients with DMD. The quality of 16 left ventricular segments in two echocardiographic views was visually graded using a binary scoring system. An endocardial border delineation percentage (EBDP) score was calculated by dividing the number of segments with adequate endocardial delineation in each imaging window by the total number of segments present in that window and multiplying by 100. Linear regression analysis was performed to model the relationship between the EBDP scores and patient age.

**Results:**

Fifty-five echocardiograms from 13 patients (mean age 11.6 years, range 3.6–19.9) were systematically reviewed. By 13 years of age, 50% of the echocardiograms were classified as suboptimal with ≥30% of segments inadequately visualized, and by 15 years of age, 78% of studies were suboptimal. Linear regression analysis revealed a negative correlation between patient age and EBDP score (*r* = −2.49, 95% confidence intervals −4.73, −0.25; *p* = 0.032), with the score decreasing by 2.5% for each 1 year increase in age.

**Conclusion:**

Echocardiographic image quality declines with increasing age in DMD. Alternate imaging modalities may play a role in cases of poor echocardiographic image quality.

## Introduction

Duchenne muscular dystrophy (DMD) is a devastating, progressive neuromuscular disease caused by mutations in the gene encoding the cytoskeletal protein dystrophin. This X-linked recessive disease is the most common inherited muscular dystrophy, affecting 1 in 3,500–6,000 live male births (1–3). Historically, progressive respiratory insufficiency led to death in almost all patients by the late second or early third decades of life. However, advances in clinical care including the use of steroids, non-invasive ventilation, and orthopedic surgery have resulted in improved survival. As a result, the prevalence of dilated cardiomyopathy and its associated morbidity and mortality have risen and Duchenne cardiomyopathy, due to progressive myocardial fibrosis and cardiac dysfunction, now accounts for 20–40% of all deaths in this population (4–6).

Subclinical cardiac dysfunction can begin as early as 6 years of age with clinical heart failure often manifesting after age 10 years and the incidence increasing with age (7). Systolic left ventricular dysfunction and ventricular remodeling is almost universally present in patients with DMD by 18 years of age (4, 7, 8). Early initiation of medical therapy, including angiotensin-converting enzyme inhibitors, aldosterone receptor antagonists, and beta receptor antagonists, may slow cardiac disease progression and remains an active area of research (9, 10). However, due to patients’ non-ambulatory status and respiratory compromise, heart failure symptoms typically do not present until advanced cardiomyopathy has developed. Routine echocardiographic surveillance of left ventricular systolic function is, therefore, recommended, starting at the time of diagnosis and continuing every 2 years until age 10 years and annually thereafter (11).

Suboptimal acoustic imaging windows caused by scoliosis, pulmonary disease, and obesity can make echocardiography technically challenging in this population. Clinical experience suggests that echocardiographic image quality declines over time, which limits accurate assessment of left ventricular function in older patients (12). Cardiac magnetic resonance imaging (CMR) has become the non-invasive gold standard for left ventricular volume and function quantification and for identifying the presence of myocardial fibrosis using gadolinium contrast administration (13–16). However, CMR is expensive and not widely available, and examinations can be lengthy and may require the use of sedation in younger children. There is a need for updated, evidence-based recommendations for left ventricular function surveillance among patients with DMD accounting for age-related factors (17).

The objectives of this study were to determine what proportion of echocardiograms in young patients with DMD demonstrate suboptimal image quality for left ventricular functional assessment and to quantify image quality deterioration with increasing patient age.

## Materials and Methods

### Participants

We performed a retrospective review of echocardiographic image quality in patients with confirmed DMD followed at a tertiary-care pediatric center (18). Echocardiograms performed using the Philips IE33 platform between January 2008 and September 2015 were included for study. This study was approved by the Conjoint Health Research Ethics Board at the University of Calgary. Patients were recruited during routine outpatient clinic visits. All patients who were able to provided written informed consent in addition to parental consent, while for younger patients unable to provide written informed consent, assent was obtained and informed consent was obtained from a parent.

### Endocardial Border Delineation Percentage (EBDP)

**Figure 1:**
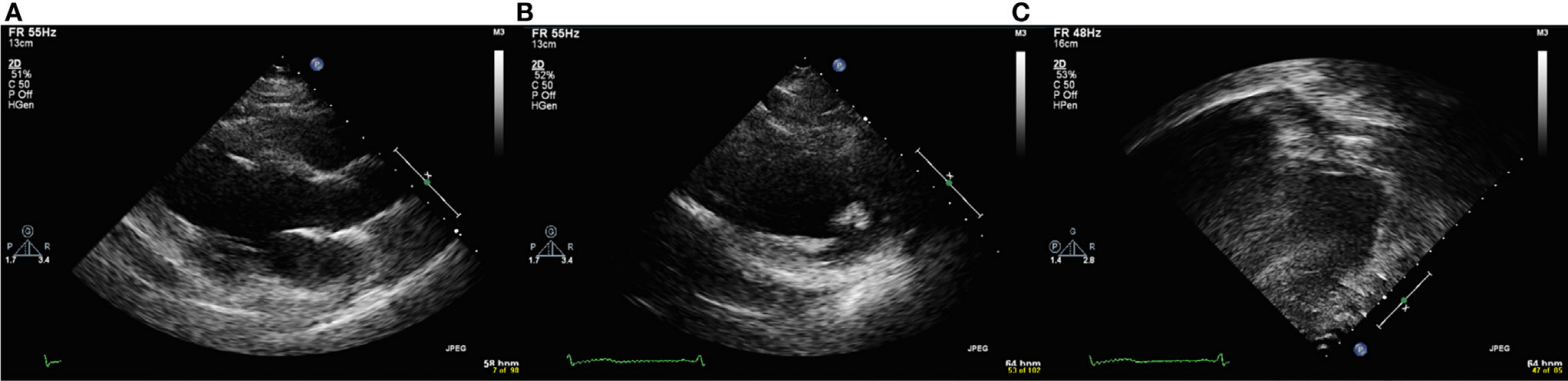
Echocardiographic images with poor endocardial border delineation from a single patient with Duchenne muscular dystrophy. **(A)** Parasternal long axis view. **(B)** Parasternal short axis view. **(C)** Apical four chamber view.

To score echocardiographic image quality, an EBDP score was calculated based on previous work (19). The American Society of Echocardiography’s 17-segment left ventricular model, excluding the apical cap, was used to score segmental image quality (20). The apical cap includes the part of the apical myocardium that is not bordered by the ventricular cavity. As the apical cap does not have an endocardial border, it was not included in our analysis. Each segment was scored in both the parasternal short axis (PSAX) views and either the parasternal long axis view or the apical 4-chamber or 2-chamber view. For each segment, the endocardial border was visually graded using a binary scoring system. A segment with adequate endocardial delineation was defined as having clear endocardial border visualization in both systole and diastole and scored “1.” All other segments were deemed as having inadequate endocardial border visualization and scored “0.” Examples of echocardiographic images with poor endocardial border delineation are shown in Figure 1.

An EBDP score was calculated by dividing the number of segments with adequate endocardial delineation in each imaging window by the total number of segments present in that window and multiplying by 100, for the following imaging windows: (1) PSAX views, (2) parasternal long axis/apical views, and (3) the total number of segments from both views (1) and (2). If an echocardiographic view was not acquired at the time of the patient’s study, then the segments from that view were excluded from analysis (rather than being coded as a “0”) so as not to artificially lower the endocardial border score. A suboptimal echocardiogram was defined as a study in which ≥30% of segments were inadequately visualized (21).

Echocardiograms were analyzed using a dedicated off-line workstation (Xcelera Cardiology Information Management version 4.1, Philips Healthcare, Amsterdam, Netherlands). Echocardiogram image quality grading was performed by a single reviewer (Sabrina Poonja). An iterative process was employed in which each echocardiogram was scored three times to account for skill improvement. By the third iteration, segment scores were being reproducibly assigned. Left ventricular ejection fraction was quantified using either the modified Simpson’s biplane method of disks (when available), or the Teichholz method. A reduced ejection fraction was defined as being ≤53% (20).

### Statistical Analysis

Data are presented as the mean (range or interquartile range) for continuous data and as frequency and percentages for categorical data. Linear regression analysis with 95% confidence intervals was performed to model the relationship between the EBDP scores and patient age. Linear regression analysis was also employed to compare patient age and EBDP scores with left ventricular ejection fraction. As multiple echocardiograms were scored for each patient, the analysis was clustered by patient using a cluster–robust covariance matrix, to account for the fact that the echocardiographic scores are independent between different patients but not necessarily independent for an individual patient. Using the Shapiro–Wilk test, we determined that EBDP scores were normally distributed when one score per patient was used. Data were non-normally distributed when all points in the data set were used (using multiple data points for each individual patient), justifying the decision to use a clustered analysis.

A *p* value of <0.05 was considered statistically significant. Assuming a SD of 20% for total endocardial border delineation score, a sample size of 42 echocardiograms would be required to have 80% power to detect a significant difference, determined to be a 20% decrease in the total endocardial border delineation score per 10-year increase in age. Analysis was performed using the statistical software package STATA, release 14 (StataCorp, College Station, Texas, USA).

## Results

### Study Population

Thirteen patients were included for retrospective assessment of echocardiographic image quality. The Table 1 displays demographic and clinical characteristics of the 13 patients. In our cohort, 23% required nocturnal non-invasive positive pressure ventilation and 38% had radiographic evidence of scoliosis and had been prescribed at least one cardiac medication by their last outpatient clinic visit. The mean number of echocardiograms reviewed for each patient was 4.23 (range 2–9). All available echocardiograms, from infancy to adolescence and young adulthood, were reviewed. The mean patient age at which echocardiograms were obtained was 11.63 years (range 3.62–19.88 years).

**Table 1:** Study patient demographics at last echocardiogram.

Patient	Age	Ambulatory status	Steroids	Cardiac medications^a^	Ejection fraction (%)^b^	NPPV	Scoliosis	Prior orthopedic surgery
1	5	EA	Yes	No	72.1	No	No	No
2	9	EA	Yes	No	65.7	No	No	No
3	10	LA	Yes	No	62.6	No	Yes	No
4	11	LA	Yes	No	57.3	No	No	Yes
5	13	EA	Yes	No	76.3	Yes	No	No
6	13	LA	Yes	No	44.4	No	No	No
7	13	NA	Yes	Yes	53.5	Yes	No	No
8	15	LA	Yes	Yes	36.9	No	No	No
9	15	NA	Yes	Yes	48.8	No	Yes	No
10	17	NA	Yes	Yes	62.7	Yes	Yes	No
11	17	NA	Yes	No	62.8	No	Yes	Yes
12	17	NA	Yes	No	61.6	No	Yes	Yes
13	20	NA	Yes	Yes	46.8	No	No	No

*EA, early ambulatory (positive Gowers’ sign, may appear clumsy, may have some difficulty with climbing, jumping, and running); LA, late ambulatory (increasing difficulty with independent ambulation, but able to walk independently); NA, non-ambulatory (unable to walk independently, rely on wheelchair for mobility); NPPV, nocturnal positive pressure ventilation*.

*^a^Cardiac medications defined as use of an angiotensin-converting enzyme inhibitor, beta blocker, or spironolactone at the last outpatient clinic visit*.

*^b^On most recent echocardiogram*.

Accurate body mass index (BMI) can become more difficult to calculate in children with DMD with increasing age due to difficulties obtaining an accurate height after patients are confined to a wheelchair. BMI information was available for 94% of children <13 years of age (31/33) and in 68% of children ≥13 years of age (15/22). Mean BMI for children <13 years of age was 20.32 (range 13.87–38.13), compared to a mean BMI for those ≥13 years of 24.64 (range 15.78–38.13).

### Endocardial Border Delineation Scoring

A total of 55 echocardiograms performed between January 2008 and September 2015 were included in the analysis. Of these studies, 21 (38%) were scored as being suboptimal (≥30% of left ventricular segments inadequately visualized). By 13 years of age, 11/22 (50%) echocardiograms were suboptimal, and by 15 years of age 7/9 (78%) were suboptimal.

The mean EBDP score from the PSAX view was 70.3% (range 0–100%, interquartile range 53.1–89.6%) and from the parasternal long axis/apical views was 79.0% (range 20–100%, interquartile range 69.3–93.8%). The mean total EBDP score was 75.6% (range 20–100%, interquartile range 65.6–88.4%).

**Figure 2:**
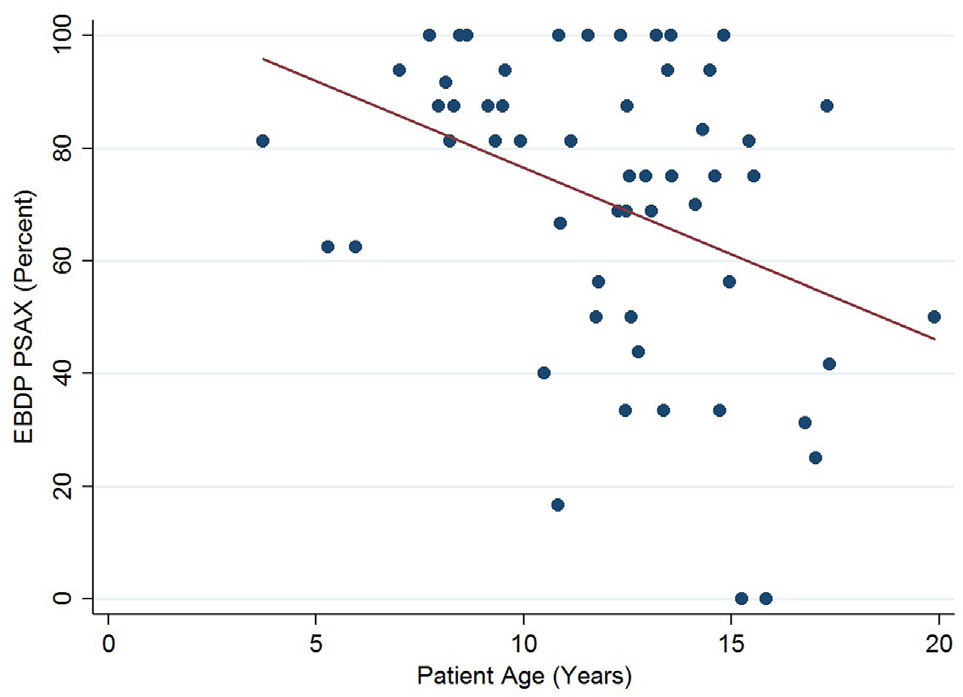
Significant negative association between endocardial border delineation percentage (EBDP) score for the parasternal short axis (PSAX) view and patient age in patients with Duchenne muscular dystrophy. Linear regression was implemented to model the relationship between EBDP PSAX and patient age (*R* = −3.08, *p* = 0.025).

**Figure 3:**
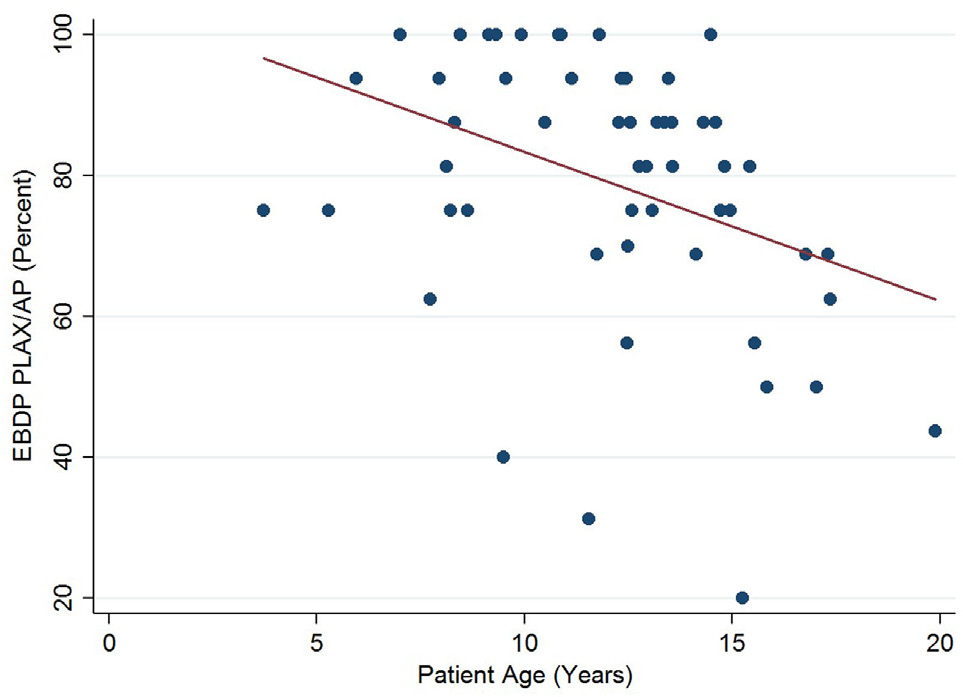
Association between endocardial border delineation percentage (EBDP) score for the parasternal long axis/apical (PLAX/AP) views and patient age in Duchenne muscular dystrophy. Linear regression was implemented to model the relationship between EBDP PLAX/AP and patient age (*R* = −2.12, *p* = 0.066).

**Figure 4:**
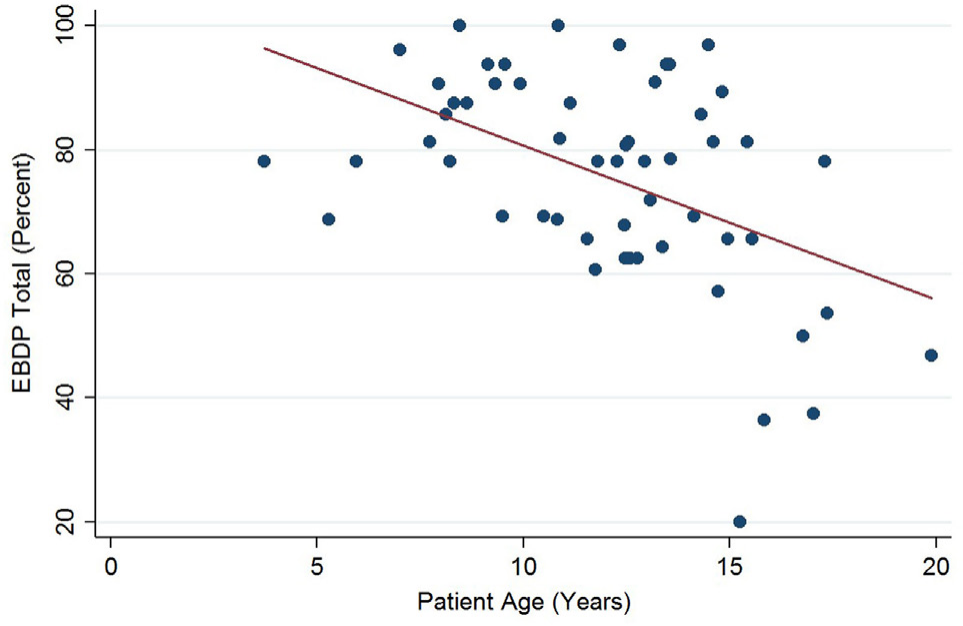
Significant negative association between endocardial border delineation percentage (EBDP) total score for all echocardiographic views and patient age in Duchenne muscular dystrophy. Linear regression was implemented to model the relationship between EBDP and patient age (*R* = −2.49, *p* = 0.032).

The relationships between the endocardial delineation scores and patient age are shown in Figures 2–4. Linear regression analysis for the PSAX view revealed a regression coefficient of −3.08 (95% confidence intervals, −5.70, −0.47, *p* = 0.025). Linear regression analysis for the parasternal long axis/apical views revealed a regression coefficient of −2.12 (95% confidence intervals, −4.41, 0.16, *p* = 0.066). Linear regression analysis for total endocardial percentage score revealed a regression coefficient of −2.49 (95% confidence intervals −4.73, −0.25, *p* = 0.032).

**Figure 5:**
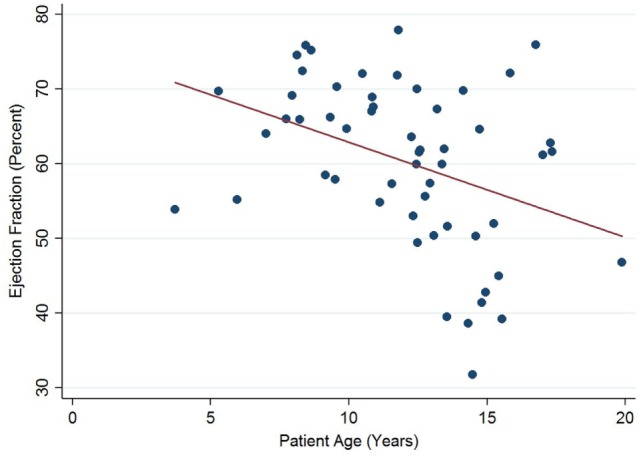
Significant negative association between left ventricular ejection fraction and patient age in Duchenne muscular dystrophy. Linear regression was implemented to model the relationship between ejection fraction and patient age (*R* = −1.27, *p* = 0.049).

Left ventricular ejection fraction was analyzed with respect to echocardiographic image quality and patient age, with a decreasing trend noted with increasing patient age (Figure 5). Linear regression analysis clustered by patient revealed a regression coefficient of −1.27 (95% confidence intervals −2.54, −0.01, *p* = 0.049). From 12 years of age onward, 16/31 (52%) of echocardiograms revealed left ventricular dysfunction (defined as ejection fraction ≤53%). There were no regional wall motion abnormalities identified in any of the echocardiograms included in this study.

## Discussion

In our cohort of pediatric patients with DMD, we demonstrate a deterioration in echocardiographic image quality with increasing patient age. There was a clinically and statistically significant decrease in echocardiographic quality with increasing age, with the total endocardial border delineation score decreasing by 2.5% for each 1 year increase in age. Previous studies have demonstrated that ejection fraction measured by echocardiography correlates poorly with CMR in patients with poor image quality (22). In our study, by 13 years of age, 50% of the echocardiograms were of suboptimal image quality, and by 15 years of age 78% of studies were suboptimal. Additionally, in our study cohort half of the echocardiograms revealed decreased left ventricular function in patients ages 12 years and older. These findings suggest that echocardiographic screening of patients with DMD in childhood may be adequate; however, by mid-adolescence, poor image quality may limit accurate assessment of cardiac function, which is important in monitoring disease progression and in evaluating the efficacy of therapy. In such cases, physicians may need to consider the role of alternate imaging modalities, such as contrast echocardiography or CMR.

The optimal timing of CMR in this population is not yet known. At our institution, we perform CMR in patients with DMD starting at age 10–12 years. Though CMR has emerged as the non-invasive gold standard for ventricular function assessment, it has important limitations that must be considered. It is expensive and not widely available. Examinations can be lengthy, which may be uncomfortable for patients with significant scoliosis. Additionally, there may be associated image artifact if metal rods have been used for spinal fusion. Finally, coordinated breathing sequences during image acquisition can be challenging for patients with more advanced respiratory involvement. Despite these limitations, in our local experience all children with DMD referred for CMR have been able to tolerate and successfully complete the study.

There may also be a role for contrast echocardiography to enhance endocardial border delineation in this patient population. Presently, use of ultrasound contrast agents is not approved for use in pediatric echocardiography. Contrast agents are used off-label for echocardiographic image enhancement in children in select centers with experience in administration to pediatric patients, although previous reports have demonstrated both safety and efficacy of ultrasound contrast in pediatric populations (23).

At our institution, our protocol is now to initiate medical therapy (i.e., angiotensin-converting enzyme inhibitor) when any abnormalities are seen by either echocardiography or CMR. However, even this may be too late given the evidence that early initiation of angiotensin-converting enzyme inhibitor therapy before the onset of measurable cardiac dysfunction is associated with delayed onset of left ventricular dysfunction and lower mortality (10). Additionally, more research is needed to determine what role advanced echocardiographic techniques, such as strain and strain rate imaging, and CMR will play in providing early markers of myocardial disease and cardiac dysfunction before a change in ejection fraction in patients with DMD, and if this information could be beneficial in guiding therapy.

Subset analysis revealed that the overall scores for the parasternal long axis and apical views were better than the PSAX views and did not deteriorate as quickly with increasing patient age. This suggests that when echocardiography is used in patients with DMD, long axis views may be considered more reliable for functional evaluation, although this is in contrast to the current guidelines for functional assessment in children, which recommend measurements in the PSAX for the calculation of ejection fraction (24, 25).

The increased mean BMI of children ≥13 years in our cohort compared to those <13 years suggests that increasing adiposity may be one of the features contributing to deteriorating image quality in this patient population. Given insufficient information available, the role of worsening scoliosis and pulmonary disease on image quality changes could not be assessed.

Other limitations for this study relate primarily to its single-center retrospective methodology and the relatively small sample size. Replication of our findings in a larger study is warranted.

## Conclusion

The high prevalence of cardiomyopathy in patients with DMD and the emerging role of early medical therapy necessitates regular and accurate cardiac screening. Though echocardiography provides useful information about left ventricular structure and function at young ages, our data quantify the decrease in echocardiographic quality with increasing patient age and highlight the limitation of echocardiography by mid-adolescence, suggesting that an alternative imaging modality, such as CMR, may be warranted. Additional research is needed to determine the optimal timing and frequency of CMR.

## Author Contributions

AP, SP, DD, KM, DP, JM, NF, and SG all contributed to the study design. SP was responsible for data acquisition. AP was responsible for statistical data analysis and for writing the manuscript. AP, SP, NF, and SG were responsible for data interpretation. All authors were responsible for critical revision of the manuscript, and approved the final version of the manuscript. AP and SP are co-first authors, with AP to be placed first.

## Conflict of Interest Statement

The authors declare that the research was conducted in the absence of any commercial or financial relationships that could be construed as a potential conflict of interest.
